# Development of data-driven modeling method for nonlinear coupling components

**DOI:** 10.1038/s41598-024-65680-3

**Published:** 2024-06-27

**Authors:** Taesan Ryu, Seunghun Baek

**Affiliations:** School of Mechanical Engineering, Pusan National University, 30 Jangjeon-Dong, Geumjeong-Gu, Busan, 46241 Republic of Korea

**Keywords:** Spars identification, Data-driven modeling, Nonlinear coupling components, Nonlinear vibration, Sponge gasket, Engineering, Materials science, Mathematics and computing

## Abstract

This research introduces a methodology for data-driven regression modeling of components exhibiting nonlinear characteristics, utilizing the sparse identification of nonlinear dynamics (SINDy) method. The SINDy method is extended to formulate regression models for interconnecting components with nonlinear traits, yielding governing equations with physically interpretable solutions. The proposed methodology focuses on extracting a model that balances accuracy and sparsity among various regression models. In this process, a comprehensive model was generated using linear term weights and an error histogram. The applicability of the proposed approach is demonstrated through a case study involving a sponge gasket with nonlinear characteristics. By contrasting the predictive model with experimental responses, the reliability of the methodology is verified. The results highlight that the regression model, based on the proposed technique, can effectively establish an accurate dynamical system model, accounting for realistic conditions.

## Introduction

In mechanical systems, components such as gaskets, mounts, washers, and O-rings play pivotal roles in mitigating vibrations and improving sealing performance. These components, fabricated from materials like rubber, metal, plastic, and foam, are instrumental in preventing leaks, aligning mechanical elements, and optimizing assembly convenience. Specifically, rubber materials, such as gaskets, mounts, and O-rings, are widely employed for enhanced sealing, vibration reduction, and shock absorption.

Rubber-like components, including gaskets, mounts, and O-rings, play a crucial role in various applications such as sealing, vibration damping, and shock absorption. These polymer materials exhibit nonlinear stress–strain behavior, influenced by strain rate variations. Exceeding stress limits can lead to plastic deformation, and prolonged loading may result in deformation over time, accompanied by the conversion of kinetic energy to thermal energy and occasional hysteresis phenomena^1–3^. Accurately modeling the mechanical properties of polymer materials is challenging due to their inherent nonlinearity and time dependence. Consequently, sophisticated analysis and modeling techniques are necessary to capture their actual behavior, prompting various studies in this field. Representative approaches encompass numerical, analytical, experimental, and data-driven methods.

The Numerical Approach provides numerically approximate solutions but lacks mathematical rigor. Luo et al.^4^ applied this method to analyze dynamic characteristics in a rubber-based rail fastener system, utilizing the superposition principle for hysteresis loop simulations on rubber components. Chen et al.^5^ extended this approach to fabric spacers, introducing asymmetric elastic force and fractional differential force. Roncen et al.^6^ expanded the Numerical Approach using the harmonic balance and shooting method, focusing on the softening effect in a rubber isolator subjected to random exciting forces.

The Analytical Approach involves solving rigorous mathematical solutions. Balasubramanian et al.^7^ used viscous damping to identify damping characteristics in rubber rectangular plates, exploring various models from energy-based to a nonlinear single-degree-of-freedom Duffing model. Mahsa et al.^8^ studied nonlinear vibration behavior in multiscale doubly curved sandwich nanoshells, grounded in Hamilton's principle. Finegan et al.^9^ explored loss characteristics in composite materials, utilizing microscopic mechanical equations, elasticity theory, and material mechanics formulas.

The Experimental Approach relies on interpreting experimental results. Kerem et al.^10^ quantified the complex modulus of a hybrid layer system, comparing model predictions with actual responses. Nagasankar et al.^11^ investigated damping effects in polymer matrix composites, analyzing the impact of fiber stacking direction and diameter. Shangguan et al.^12^ constructed models for nonlinear rubber torsional vibration absorbers, exploring various models such as the Maxwell model, Voigt model, and fractional derivative model based on experimental results.

The Data-Driven Approach accesses system data through a processing system. Conti et al.^13^ integrated Finite Element Method (FEM) and SINDy to construct a Reduced Order Model (ROM) with an encoder neural network. Brunton et al.^14^ extended the SINDy method to macroscopic modeling studies on metamaterials. Wang et al.^15^ explored the relationship between microstructure and mechanical properties in polymer nanocomposites using a data-driven deep learning approach. Kazi et al.^16^ used the SINDy method to predict strain–stress curves of composite materials with consistent mechanical behavior. These approaches, combining microscopic insights with data-driven modeling, contribute to understanding the complex behaviors exhibited by polymer materials. In contemporary research endeavors, there is a growing trend to employ Sparse Identification of Nonlinear Dynamics (SINDy) for modeling nonlinear systems, accompanied by a parallel exploration of extensive data pertaining to regression targets. However, the challenges inherent in acquiring sufficient empirical data for regressing real-world systems pose a formidable obstacle. This limitation results in numerous regression models failing to adeptly capture the dynamic nuances of the actual system, particularly when confronted with subtle changes in system conditions.

This research introduces an innovative approach designed to enhance SINDy through a simplified process, addressing the pervasive issue of insufficient data. It integrates dynamic background knowledge with data regression techniques. Typically, when analyzing nonlinearity through data-driven methods, only data are often utilized. This leads to high accuracy in the regression domain but poses accuracy challenges in other areas. Therefore, this study innovatively implements constraints representing the dynamics of the physical domain during data regression. This strategically handles the generation of comprehensive data sets for real systems by integrating parameters characterized by linear relationships into the regression variable set. This method strategically addresses the generation of comprehensive datasets for real systems by incorporating parameters characterized by linear relationships into the set of regressors. Notably, the intentional weighting of linear terms enhances the extraction of models that are both more general and realistic, even when confronted with limited empirical data. To assess the effectiveness of this novel method, it is applied to a model describing the equation of motion for a vibrating gasket system. The outcomes of this application underscore the reliability of the proposed approach in accurately predicting the response of nonlinear vibration systems. A distinctive aspect of note is the method's ability to identify real systems without necessitating intricate material complexity or extensive mathematical analyses during the model construction phase. A noteworthy characteristic of this approach is the intentional fixing of weights on linear terms, contributing to heightened efficiency in system identification even with a relatively modest amount of available data. The resultant models, derived through this methodology, exhibit a commendable capacity to effectively track system responses, even in non-regressive conditions. This underscores the pragmatic utility of the proposed approach, particularly in scenarios characterized by limited data availability.

## Methodology

This chapter outlines the methodology employed in utilizing the SINDy method to model a nonlinear fastening component within a system. The objective is to estimate the governing equations that describe the behavior of this component. The process involves the application of an algorithm for sparse regression modeling, where weights are introduced to linear terms, and an error histogram facilitates the extraction of a robust model. This method proves particularly advantageous for nonlinear vibration systems prone to model divergence, which often hampers the accurate tracking of actual behavior. By acting as a preventive measure, this approach contributes to the development of more generalized models compared to traditional methods.

The research focus centers on elucidating the nonlinear equations governing the dynamic motions of coupling components, a notably intricate aspect of system dynamics. To assess the nonlinear behavior of an unidentified component, a meticulously designed experimental apparatus was employed. Data obtained from system identification experiments were utilized to construct three regression models. The first model, Case 1, assumed linearity and was created through linear regression to establish a baseline control group. To capture the system's nonlinearity, two sparse regression models, Case 2 and Case 3, were developed using techniques such as error histogram and L1 regularization. These models were separately regressed, with Case 3 incorporating weights assigned to linear elements, offering diverse perspectives on addressing the system's nonlinearity.

The validation process for the regression models entailed designing a separate validation system characterized by diverse physical conditions in comparison to those of the identification system. The reliability of the modeling process was affirmed by comparing the validation system's response to predictions from the three regression models (Cases 1, 2, 3) individually. This chapter provides an in-depth exploration of the theoretical background and methodology employed in the regression modeling of the nonlinear system, encompassing both the experimental setup for identification and the subsequent validation process.

### Theorical background

SINDy, a data-driven technique, serves the purpose of estimating dynamic system governing equations from observational data, particularly in cases where modeling proves challenging or unknown. By collecting time-varying data and integrating them with measured system characteristics, SINDy utilizes sparse regression analysis, with a specific focus on Lasso regression, to identify the optimal model. This robust algorithm directly estimates governing equations for nonlinear systems from data, proving especially beneficial in scenarios where dynamic modeling presents challenges^17,18^.

To derive governing equations from data, time history data of the subject (${\varvec{Y}}$) and its parameters (${\varvec{X}}$) evolving over time were systematically gathered. A library, denoted as $\boldsymbol{\Theta }({\varvec{X}})$, comprised potential functions correlating with the subject, utilizing the collected data. Following the selection of suitable functions for the library, a relationship emerged between the subject and the library, expressed by a coefficient vector $\boldsymbol{\Xi }$, satisfying Eq. (1):

1 $${\varvec{Y}}=\boldsymbol{\Theta }({\varvec{X}})\boldsymbol{\Xi }$$

The coefficient vector $\boldsymbol{\Xi }$ signifies the connection between ${\varvec{Y}}$ and $\boldsymbol{\Theta }({\varvec{X}})$, revealing the active functions from the candidate functions in the library. To prevent overfitting, it is assumed that only a selected subset of candidate functions holds significance in representing ${\varvec{Y}}$, thereby framing the issue as a sparse regression problem. This approach aims to identify the activated column vectors in the library. The sparse coefficient vector ($\boldsymbol{\Xi }$) denotes which candidate functions are active and their respective coefficients. The derivation of the coefficient vector $\boldsymbol{\Xi }$ involves the utilization of the Moore–Penrose pseudo-inverse matrix, as articulated below:

2 $$\boldsymbol{\Xi }={\boldsymbol{\Theta }}^{+}({\varvec{X}}){\varvec{Y}}$$

The Moore–Penrose pseudo-inverse matrix yields a dense vector $\boldsymbol{\Xi }$ with the minimum norm. To induce sparsity, employ techniques like lasso regression, imposing constraints by assuming variables with low linear dependence as 0. This involves minimizing the regularization cost, denoted as Eq. (3), where the regularization parameter $w$ determines the weight assigned to sparsity. Through this approach, achieve sparse regression modeling to obtain ${\boldsymbol{\Xi }}_{{\varvec{i}}}$. In Eq. (3), the coefficient $w$(sparsification parameter) is a variable associated with sparsity and complexity. A higher value of $w$ emphasizes sparsity over complexity, whereas a lower value of $w$ emphasizes complexity over sparsity. Thers is no precise mathematical equation for determining $w$, however, it would be helpful to determine $w$ value through cross-validation using machine learning^17,18^.

3 $${\boldsymbol{\Xi }}_{{\varvec{i}}}=\mathit{arg}\underset{{\boldsymbol{\Xi }}_{{\varvec{i}}}^{\boldsymbol{^{\prime}}}}{\mathit{min}}{\left|\left|{\varvec{Y}}-\boldsymbol{\Theta }\left({\varvec{X}}\right){\boldsymbol{\Xi }}_{{\varvec{i}}}^{\boldsymbol{^{\prime}}}\right|\right|}_{2}+w{\left|\left|{\boldsymbol{\Xi }}_{{\varvec{i}}}^{\boldsymbol{^{\prime}}}\right|\right|}_{1}$$

### Regression algorithm with linear weightening

**Figure 1:**
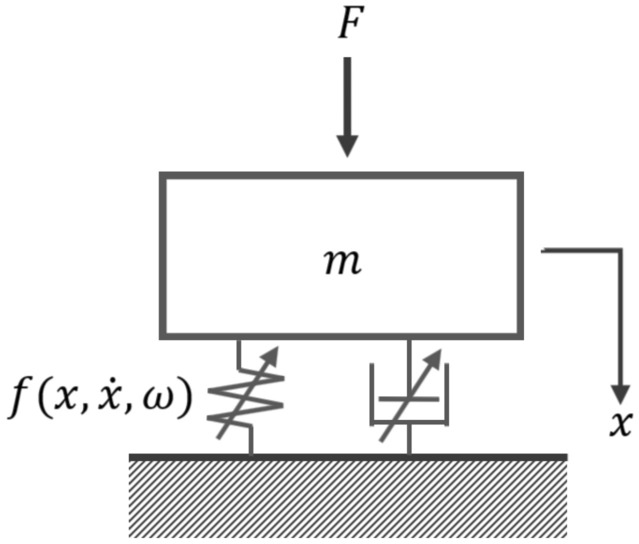
Single-degree-of-freedom vibration system schematic.

The general problem treated here is schematically depicted in Fig. 1. A single-degree-of-freedom (SDOF) vibration system with nonlinear component is depicted in Fig. 1. The equation governing the SDOF system is expressed as:

4 $$F - m\ddot{x} = f\left(x,\dot{x},\omega \right)$$

Here, $F$ represents the applied force, $f$ denotes the nonlinear force, $m$ is mass, $x$ represents the displacement, and $\dot{x}$ and $\ddot{x}$ are the first and second derivatives of displacement, respectively. Additionally, $\omega$ denotes applied frequency. In practical applications, it is straightforward to discern the characteristics of solid components characterized by high stiffness. However, interpreting the dynamic behavior of connecting components with made of soft material proves to be challenging. The direct measurement of these components is hindered by the absence of distinct material points or designated measurement points. Consequently, the measurement of the nonlinear force is indirectly conducted from the sensor point on the solid components due to these constraints.

**Figure 2:**
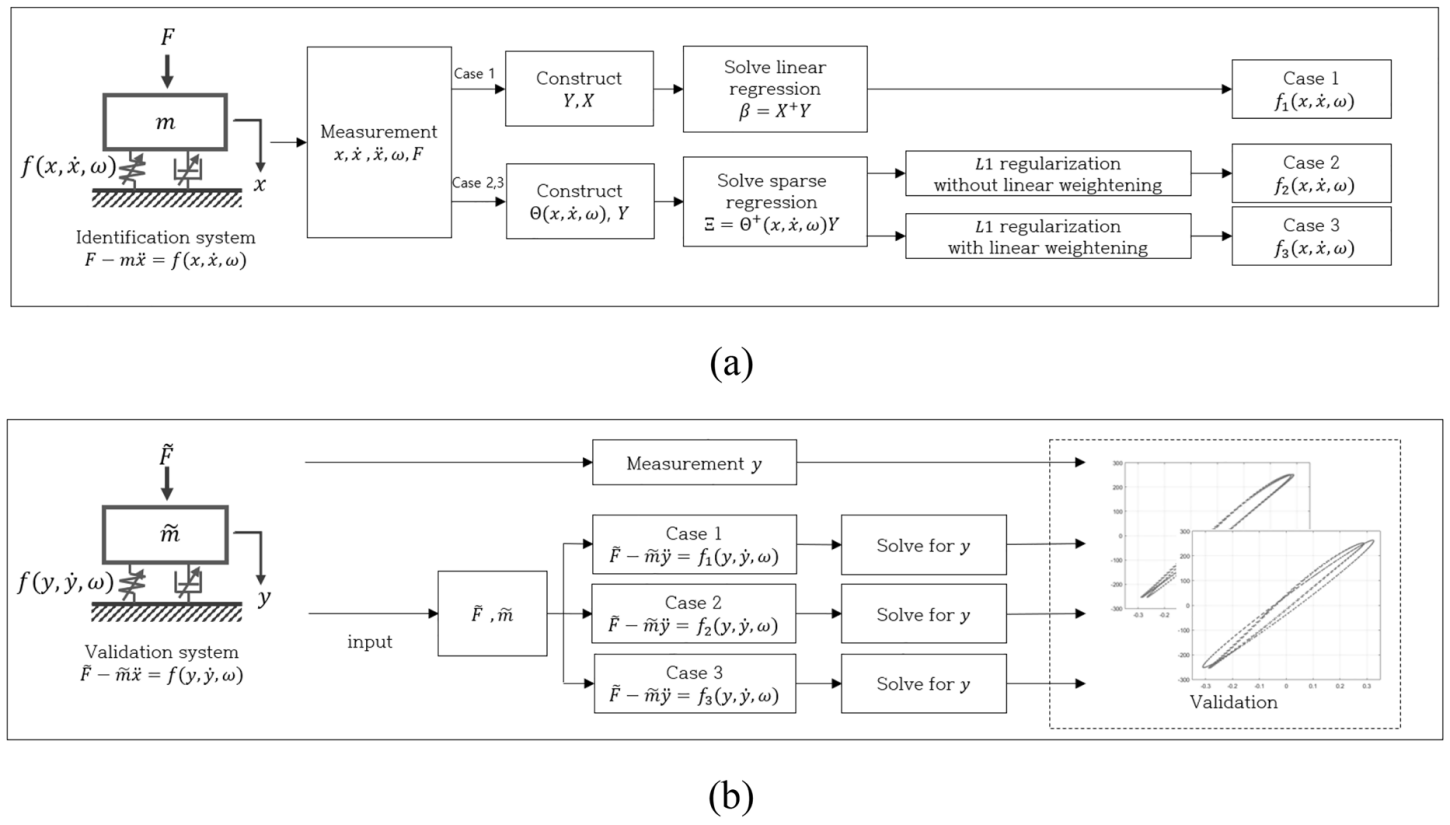
Flowchart of (**a**) identification process of nonlinear component and (**b**) validation process.

The conventional SINDy approach presents challenges in identifying a practical expression for the nonlinear force. In response to this limitation, our study introduces an effective linear weighting process. To assess the efficacy of this proposed process, we examine three regression models focused on identifying a single-degree-of-freedom vibration system with a nonlinear force. Extracting time history data $(x, \dot{x}, \ddot{x}, \omega , F)$ from the identification system, three models emerge: a linear model (Case 1), a sparse model without linear weights (Case 2), and a sparse model with linear weights (Case 3), as detailed in Fig. 2a. The reliability of these models is validated by predicting system responses to variations in mass within the identification system. The evaluation entails calculating errors based on both least square and peak-to-peak criteria, as illustrated in Fig. 2b. This approach allows for a comprehensive assessment of the proposed weighting process and its impact on the accuracy of the identified models.

**Figure 3:**
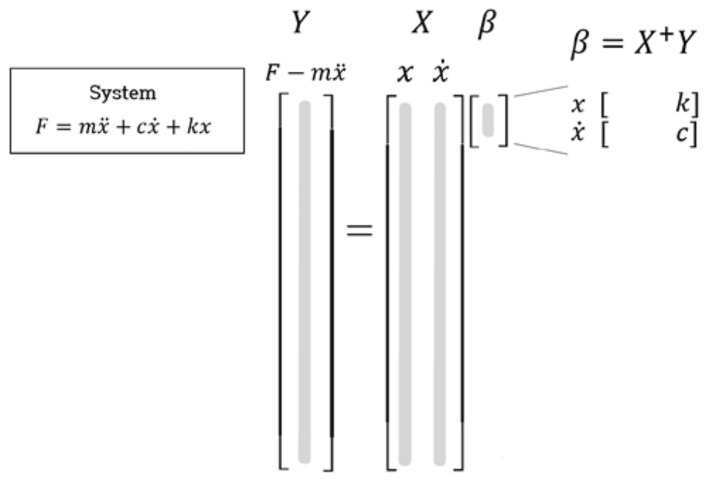
Linear regression configuration.

The linear vibration model, denoted as Case 1, is formulated by employing constant damping ($c$) and a stiffness ($k$) coefficient. Consequently, the physical interpretation of the force from the component can be expressed as $f\left( {x,\dot{x},\omega } \right) = c\dot{x} + kx$. To ascertain the values of the damping and stiffness coefficients, linear regression is conducted, as illustrated in Fig. 3. By taking the Moore–Penrose pseudo-inverse matrix of $X$, the coefficient vector $\beta$ can be determined. In many instances, this problem is over constrained, leading to coefficients that remain invariant with respect to the excitation frequency ($\omega$). This observation underscores the inefficacy and instability of the linear regression approach in identifying the damping and stiffness characteristics of the system under consideration.

**Figure 4:**
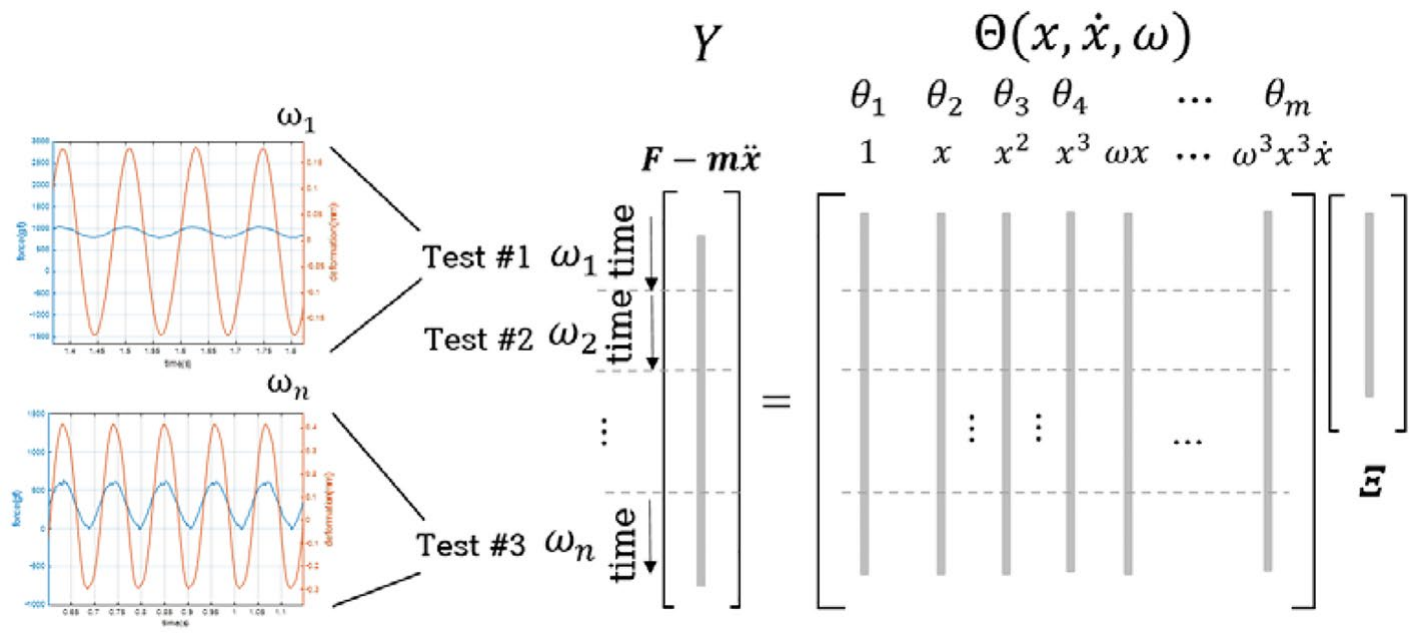
Library stack configuration.

Sparse regression models (Case 2 and Case 3) were devised using the SINDy approach. Similar to the linear model, the time-varying dependent variable vector $Y$ is defined as $F - m\ddot{x},$ where $F$ represents the external vibration force, and $m\ddot{x}$ is the inertia force. The nonlinear coefficients, specifically stiffness and damping, may vary based on factors such as boundary force, deformation, deformation rate, and excitation frequency. Consequently, library functions are meticulously chosen depending on the values of $x, \dot{x},$ and $\omega$. In the pursuit of creating a comprehensive sparse regression model, a diverse range of frequencies was systematically integrated into the library, as illustrated in Fig. 4. The regression target $Y$ and the library matrix $\Theta$ were organized as cumulative data collected at multiple excitation frequencies. This methodology streamlines the development of a modeling approach capable of capturing the system's response across a broad spectrum of frequency conditions.

**Figure 5:**
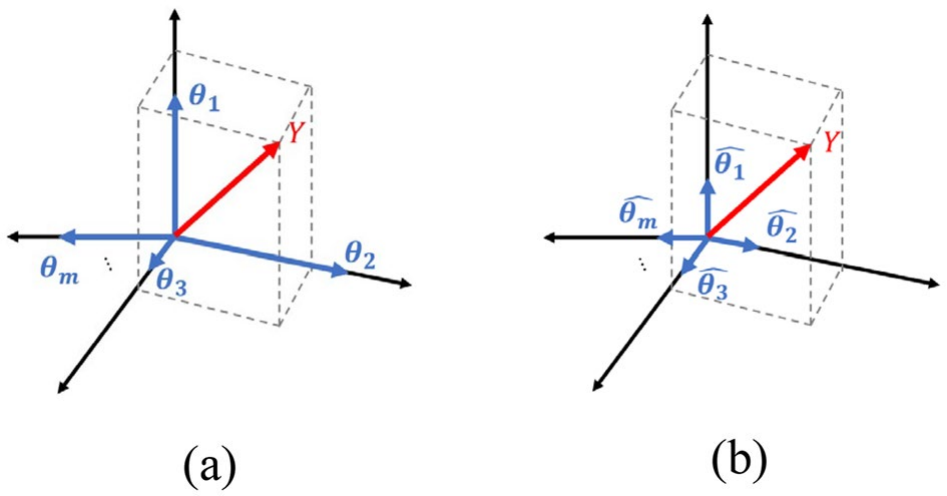
Normalizing library column vectors: (**a**) Unnormalized coordinate system and (**b**) Normalized coordinate system.

During the library construction process, the inclusion of physical quantities is influenced by units and dimensions, impacting the column vector's norm. To ensure accurate derivation of governing equations, all library column vectors underwent normalization, as illustrated in Fig. 5. In Fig. 5a, an unnormalized coordinate system for parameter vectors is depicted, introducing scaling issues that can disproportionately affect a large norm vector. Consequently, to address concerns related to vector scaling, all parameter vectors were normalized as shown in Fig. 5b. Throughout the normalization process, adjustments were made to accommodate any changes in vector norm size after model regression.

Theoretical expectations dictate that the library's column vectors should exhibit sparse dependence on the recursion target. However, overfitting may manifest due to insufficient or noisy accumulated data, or if the library is inadequately chosen. Additionally, in oscillatory systems, the damping value is influential in the convergence of the system, but if its magnitude is small, it may not be able to be identified effectively. This paper addresses these challenges by deliberately assigning weights to linear stiffness and linear damping, corresponding to Case 3, as detailed in Algorithm 1. Step 1 involves normalizing the library column vector for accurate sparse regression. Corrections to the changing norm size occur in Step 3. Step 2 encompasses the sparse regression process, where the constraint value $\lambda$ adapts based on the number of parameters ($n$), and the model is extracted assuming that parameters larger than the $\lambda$ value are linearly dependent. In this process, the difference between Case 2 and Case 3 is confirmed. Unlike Case 2, Case 3 receives the linear terms index included in the library and assumes that it is always included in the regression model. This was done by maximizing the elements value of the linear terms in the dense coefficient vector ${\boldsymbol{\Xi }}_{{\varvec{d}}{\varvec{e}}{\varvec{n}}{\varvec{s}}{\varvec{e}}}$. This is expressed as ${\boldsymbol{\Xi }}_{{\varvec{d}}{\varvec{e}}{\varvec{n}}{\varvec{s}}{\varvec{e}}}(m)=\text{max}\left(abs\left({\boldsymbol{\Xi }}_{{\varvec{d}}{\varvec{e}}{\varvec{n}}{\varvec{s}}{\varvec{e}}}\right)\right)$ in Algorithm 1.

**Algorithm 1**: model regression algorithm for Case 2, 3
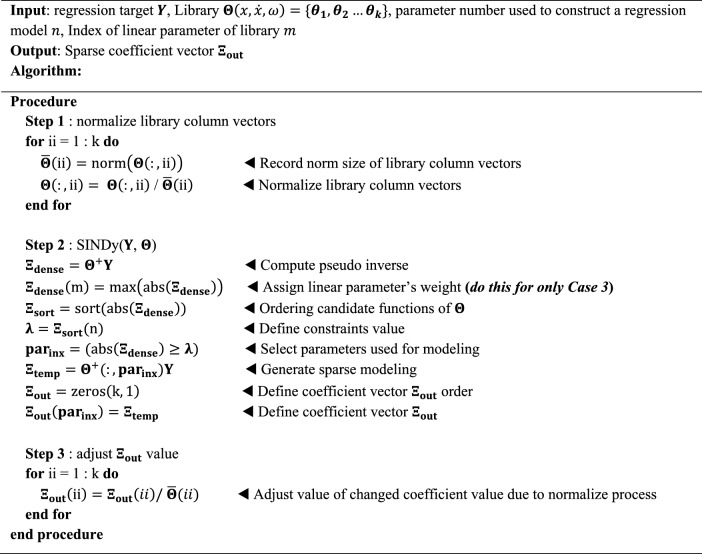


### Experiment setup

**Figure 6:**
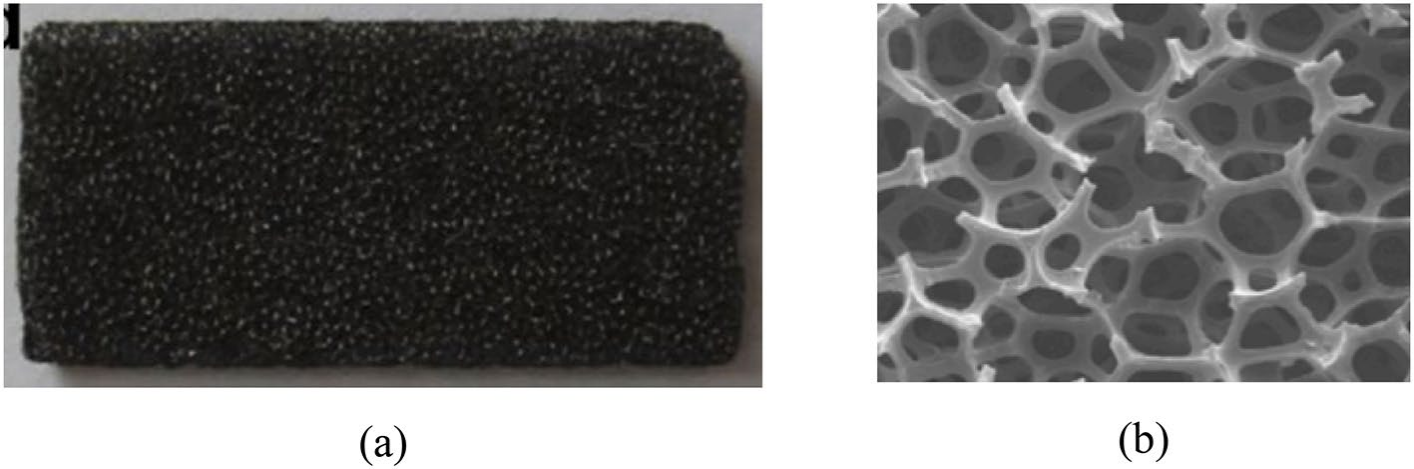
Geometry of sponge^21^: (**a**) The photograph of porous sponge and (**b**) The microscopic structure of porous sponge.

**Figure 7:**
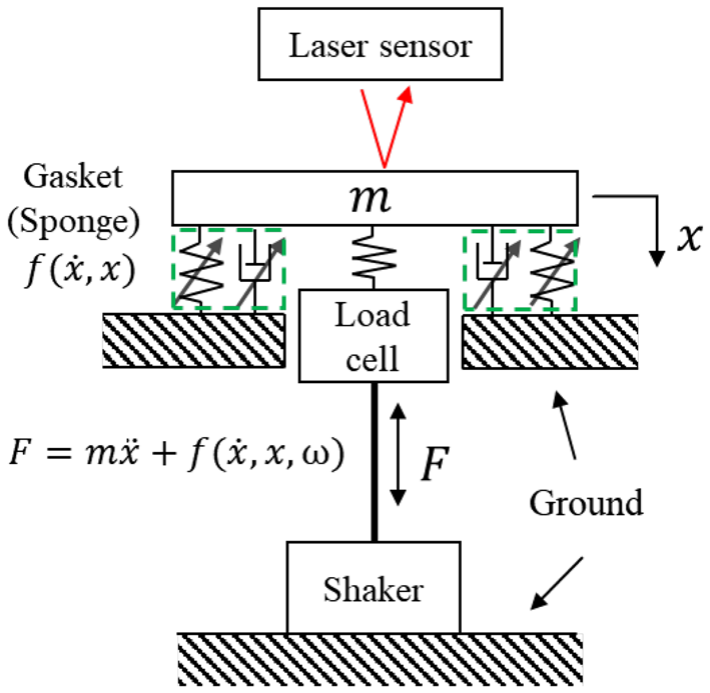
Schematics of SDOF nonlinear vibration system.

This section introduces the experimental setup designed to assess the dynamic characteristics of nonlinear materials, exemplified by sponges. Figure 6 showcases the material employed in the experiment. In Fig. 6b, a scanning electron microscope (SEM) provides a detailed depiction of the microscopic features of the sponge. The intricate porous geometry, characterized by numerous tiny pores exhibiting irregular patterns, poses a challenge for mathematical modeling due to its geometric complexity. In this regard, Nie et al.^19^ studied the behavior of the porous structure through numerical methods, and it can be confirmed that nonlinearity of porous structures using FEM. Liu et al.^20^ studied a methodology for predicting nonlinear behavior of porous and heterogeneous structures. From through thesis, it can be estimated that the porous structure has non-linearity, so the experiment was performed with the sponge set as non-linear. To probe the nonlinear behavior of sponge-like materials, a gasket-fastened vibration system is employed, as depicted in Fig. 7. The schematic illustrates a 1-D vibration system where the shaker is securely affixed to the ground, ensuring that the excitation is exclusively transmitted to the mass. This mass is connected to the sponge, which is fixed to the ground. The displacement of the mass is precisely measured using a laser sensor, offering a resolution level of 0.01 µm. The utilization of this vibration system aids in investigating and understanding the complex nonlinear dynamics inherent in sponge materials.

**Figure 8:**
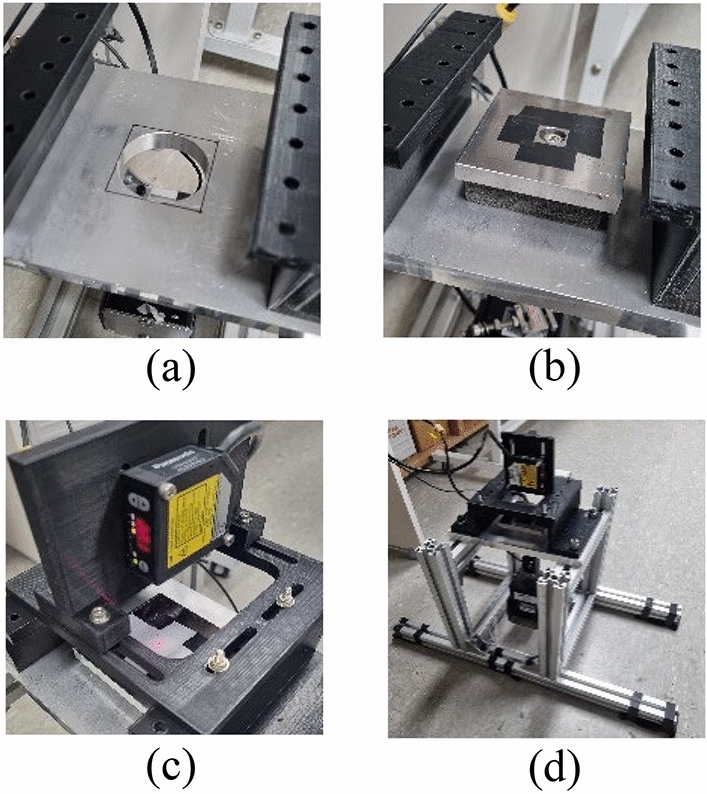
Experiment setup: (**a**) ground plate, (**b**) mass and gasket, (**c**) displacement measurement laser sensor and (**d**) overall experiment design.

A detailed depiction of the experimental setup is presented in Fig. 8. Figure 8a showcases an aluminum ground plate with a central hole and bolting holes on each side, firmly secured by bolts. In Fig. 8b, a concentrated mass is affixed to the ground alongside a rectangular prism-shaped sponge gasket, featuring a central square hole for the bolt. The load cell establishes a connection between the concentrated mass, the holes in the sponge gasket, and the ground. Figure 8c exhibits a laser sensor responsible for measuring displacement, while Fig. 8d provides an overview of the entire experimental arrangement. Maintaining consistent contact conditions for the sponge gasket involved securely fixing the mass ($m$) in contact with the ground boundary. To prevent the detachment of the concentrated mass, a pre-load was applied, compressing the gasket to achieve a thickness of approximately 10 mm. The amplification of the applied force was facilitated through the voltage gain of a non-inverting amplifier utilizing an operational amplifier (OP-AMP, LM324). Signal measurement was conducted using an oscilloscope (TDS1002B). Comprehensive details regarding the experiment's structures and sensors are presented in Table 1.

**Table 1:** Identification experiment configuration.

Components	Dimensions	Remark & mass
sponge gasket	W (65 mm) H (65 mm) T (18 mm)	pre-load, 10 mm
lumped mass	W (80 mm) H (80 mm) T (10 mm)	steel (500 g)
load-cell	–	Bongshin loadcell inc. DBCM-10 model (50 g)
ground	T (15 mm) plate	Aluminium center-hole $\phi$ 50 mm
laser sensor	–	Panasonic inc. HL-G105-S-J model

**Figure 9:**
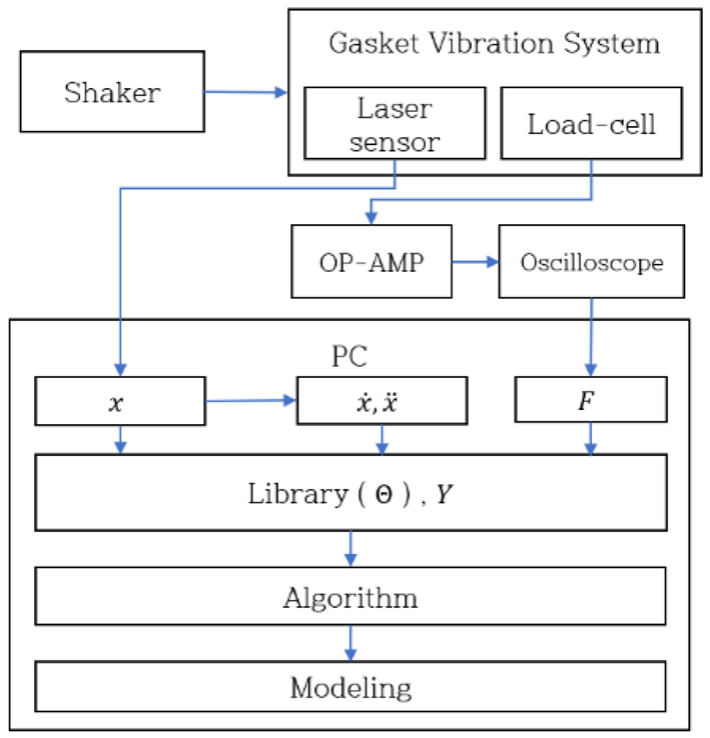
Process of SDOF nonlinear vibration system identification.

As depicted in Fig. 9, the experimental procedure involved the application of an excitation force to the concentrated mass within the gasket system using a shaker. During steady-state vibration, both displacement and excitation force of the concentrated mass were concurrently measured. This was achieved through the utilization of a laser sensor for displacement data and a load cell for capturing the excitation force. The displacement data, acquired from the laser sensor, served as the basis for determining velocity and acceleration. To consider nonlinearity effectively, the sampling frequency of the laser sensor and load cell was sustained several tens of times higher than excitation frequency. The excitation frequency was below 12 Hz, the laser sensor was measured up to 750 Hz, and the load cell was measured below a frequency of 500 Hz.

The collected data underwent thorough processing through a specialized algorithm designed to build a comprehensive library and formulate a robust regression model. This systematic approach allowed for a detailed analysis of the dynamic characteristics of the sponge material under the influence of excitation forces.

### Validation test

**Figure 10:**
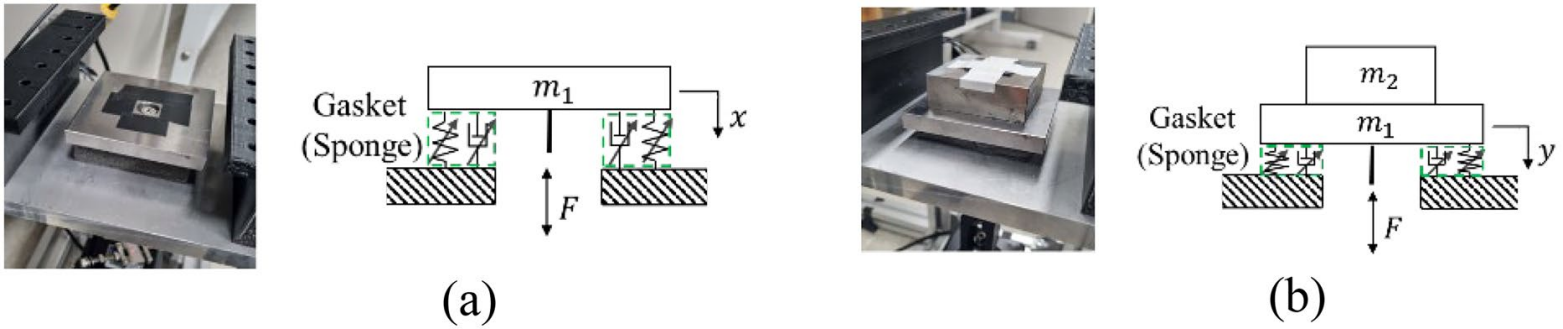
Schematics of identification system and validation system: (**a**) Identification system and (**b**) Validation system.

The validation system was meticulously constructed through modifications to the mass of the identification system. Illustrated in Fig. 10, an additional mass, denoted a ${m}_{2}$, was introduced to the validation system, serving to differentiate its configuration from that of the identification system. This deliberate adjustment in the system's weight introduces distinguishable changes in both the inertia force and the nonlinear reaction emanating from the sponge gasket. The addition of ${m}_{2}$ in the validation system provides a controlled variation, allowing for a nuanced examination of how alterations in mass influence the dynamic response of the sponge material. This intentional manipulation of the system's weight serves as a crucial component in validating the robustness and generalizability of the identified characteristics obtained from the identification system. The subsequent analysis of these changes contributes to a comprehensive understanding of the nonlinear behavior exhibited by the sponge material under varying experimental conditions.

## Result

The reliability of the proposed methodology is substantiated through the thoughtful design and execution of both identification and validation systems. In the identification experiment, the model adeptly captures the nuanced characteristics of stiffness and damping exhibited by the sponge gasket. To corroborate the robustness of the methodology, validation experiments were conducted, involving a meticulous comparison between the measured responses and the predicted responses. This systematic validation process serves as a critical step in ensuring the model's accuracy and applicability under varying conditions. The assessment of the utility of a regression model with fixed linear weights forms a pivotal aspect of the validation process. Specifically, the accuracy of three distinct responses is comprehensively compared, shedding light on the model's predictive capabilities. Additionally, the validation extends to a scrutiny of convergence/divergence issues and a thorough comparison of both least squares and peak-to-peak errors. These comprehensive validation measures collectively contribute to affirming the reliability and efficacy of the proposed methodology in capturing and predicting the dynamic behavior of the sponge material.

### Identification experiment results

The gasket vibration system manifests a dual nature, embodying characteristics of both a linear mass-stiffness-damper system and a mass-nonlinear stiffness-nonlinear damper system. To systematically investigate these attributes, the identification system's mass was precisely set at 500 g. Utilizing a sinusoidal force as the excitation, the identification system was subjected to a comprehensive range of excitation frequencies spanning from 1 to 10 Hz, covering a total of 18 frequencies. Throughout this experimental spectrum, measurements of responses and applied forces were meticulously recorded. Vibration displacement, ranging between − 0.8 mm and 0.8 mm, corresponded to a strain ($\varepsilon$) magnitude spanning from − 0.08 to 0.08.

**Figure 11:**
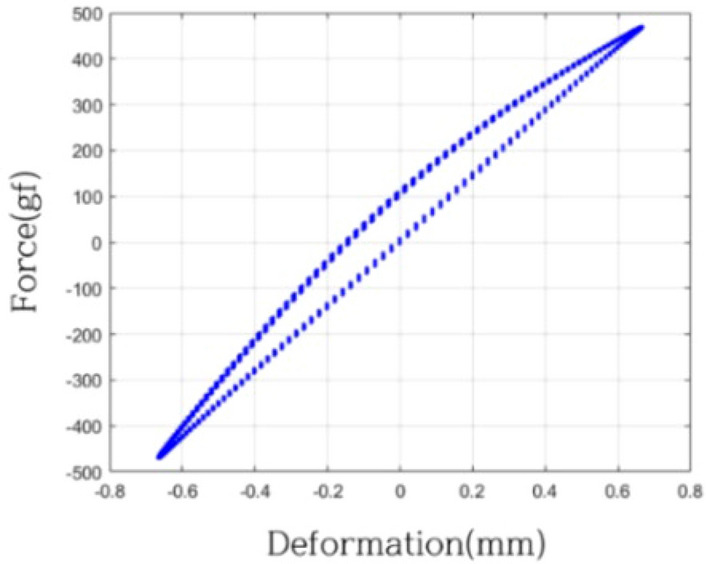
Deformation-force curve of identification system (5.4 Hz).

The resulting displacement-force curve, illustrated in Fig. 11, serves as a graphical representation of the system's nonlinearity. Notably, variations in damping sizes during gasket extension and contraction are evident, affirming the nuanced and nonlinear dynamics inherent in the gasket vibration system. This experimental approach contributes to a comprehensive understanding of the system's behavior under diverse excitation conditions, crucial for accurate modeling and analysis.

Utilizing $F-m\ddot{x}$ as the dependent variable and $x$ and $\dot{x}$ as independent variables, the linear model (Case 1) was regressed through linear regression. Equation (5) and Table 2 present the equation of motion and coefficients of the model.

**Table 2:** Regression modeling for Case 1.

Coefficient	Value (unit: g, mm, s)
${k}_{0}$	7.8632 $\times {10}^{6}$
${c}_{0}$	2.2621 $\times {10}^{4}$

5 $$F-m\ddot{x}={k}_{0}x+{c}_{0}\dot{x}$$

Initiating the sparse regression model (Case 2, 3), the library's column vectors underwent normalization. Sequentially, linear regression applied the Moore–Penrose pseudo-inverse matrix to regress $Y$. This yielded the dense coefficient vector ${\Xi }_{dense}$, defining candidate functions and their respective weight assignments. The resulting ${\Xi }_{dense}$ is detailed in Table 3. By sorting the dense coefficient vector ${\Xi }_{dense}$ by its elements absolute magnitude, the parameters affecting the system are identified. As a result, multiple regression models were constructed, incorporating weights that not only reflected the magnitude of correlation but also considered the number of parameters utilized in the analysis. Various modeling scenarios were executed, varying the number of candidate functions in the regression model. Evaluation of model appropriateness involved calculating the error between the measured response (${Y}_{test}$) and the predicted model response (${Y}_{model}$). This facilitated the identification of the optimal number of candidate functions required for modeling. The prediction model's error was determined through comparing the norm of the time series vector ${Y}_{test}-{Y}_{model}$ with the norm of the time series vector ${Y}_{test}$, as represented in Eq. (6).

**Table 3:** Linear regression coefficient vector of system.

Candidates	Value	Candidates	Value
1	− 0.2107 $\times {10}^{8}$	$\dot{x}$	2.2998 $\times {10}^{8}$
$x$	3.8049 $\times {10}^{8}$	$x\dot{x}$	0.0092 $\times {10}^{8}$
${x}^{2}$	− 0.0683 $\times {10}^{8}$	${x}^{2}\dot{x}$	1.2828 $\times {10}^{8}$
${x}^{3}$	− 1.1669 $\times {10}^{8}$	${x}^{3}\dot{x}$	0.4529 $\times {10}^{8}$
$\omega$	1.2179 $\times {10}^{8}$	$\omega \dot{x}$	− 5.2913 $\times {10}^{8}$
$\omega x$	− 2.0121 $\times {10}^{8}$	$\omega x\dot{x}$	− 0.1402 $\times {10}^{8}$
$\omega {x}^{2}$	0.0892 $\times {10}^{8}$	$\omega {x}^{2}\dot{x}$	− 4.3672 $\times {10}^{8}$
$\omega {x}^{3}$	3.3597 $\times {10}^{8}$	$\omega {x}^{3}\dot{x}$	− 1.2868 $\times {10}^{8}$
${\omega }^{2}$	− 1.3658 $\times {10}^{8}$	${\omega }^{2}\dot{x}$	5.6509 $\times {10}^{8}$
${\omega }^{2}x$	3.3236 $\times {10}^{8}$	${\omega }^{2}x\dot{x}$	0.2317 $\times {10}^{8}$
${\omega }^{2}{x}^{2}$	− 0.1002 $\times {10}^{8}$	${\omega }^{2}{x}^{2}\dot{x}$	4.7960 $\times {10}^{8}$
${\omega }^{2}{x}^{3}$	− 3.9071 $\times {10}^{8}$	${\omega }^{2}{x}^{3}\dot{x}$	1.2199 $\times {10}^{8}$
${\omega }^{3}$	0.4798 $\times {10}^{8}$	${\omega }^{3}\dot{x}$	− 2.3018 $\times {10}^{8}$
${\omega }^{3}x$	− 1.3974 $\times {10}^{8}$	${\omega }^{3}x\dot{x}$	− 0.1096 $\times {10}^{8}$
${\omega }^{3}{x}^{2}$	0.0011 $\times {10}^{8}$	${\omega }^{3}{x}^{2}\dot{x}$	− 1.7529 $\times {10}^{8}$
${\omega }^{3}{x}^{3}$	1.4670 $\times {10}^{8}$	${\omega }^{3}{x}^{3}\dot{x}$	− 0.3778 $\times {10}^{8}$

6 $$error=\frac{|{{\varvec{Y}}}_{{\varvec{t}}{\varvec{e}}{\varvec{s}}{\varvec{t}}}-{{\varvec{Y}}}_{{\varvec{m}}{\varvec{o}}{\varvec{d}}{\varvec{e}}{\varvec{l}}} |}{\left|{{\varvec{Y}}}_{{\varvec{t}}{\varvec{e}}{\varvec{s}}{\varvec{t}}}\right|}$$

**Figure 12:**
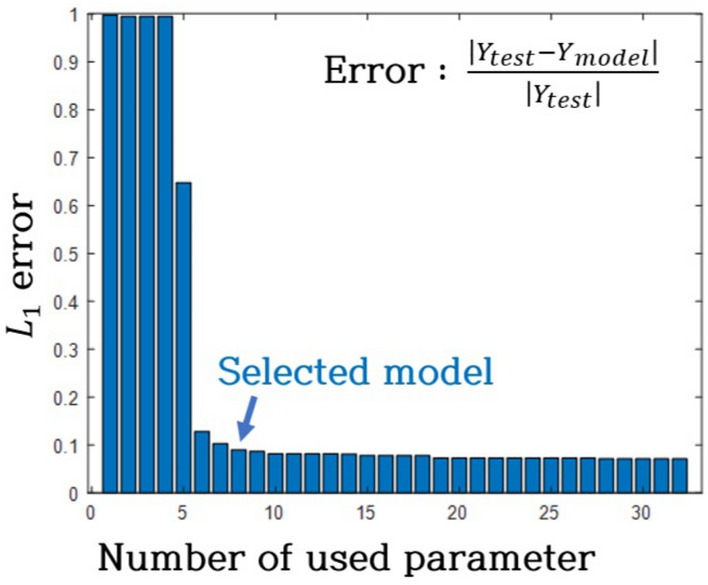
Optimized model selection from error histogram.

**Figure 13:**
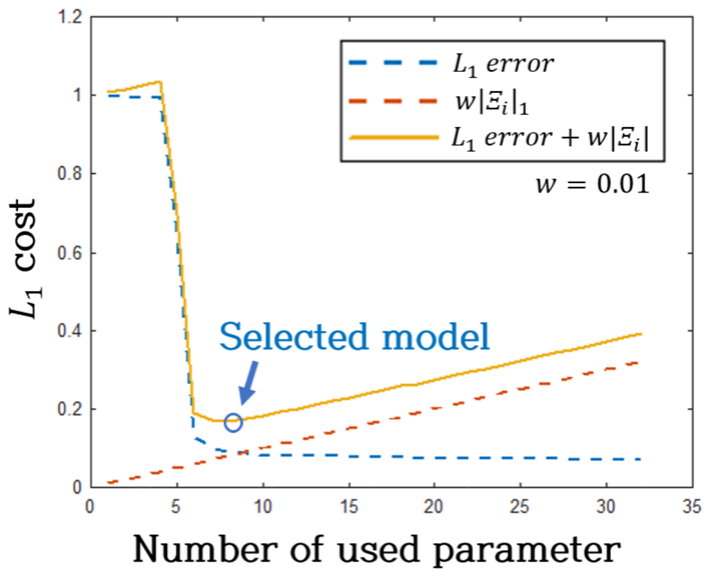
L1 cost from error histogram.

The ${L}_{1}$ error trend, depicted in Fig. 12, was explored by varying the number of candidate functions in the modeling process. Figure 13 illustrates the ${L}_{1}$ cost function, incorporating error and sparsity for optimal model selection. In Fig. 13, the solid line represents the cumulative cost of the model, comprising error and sparsity costs. In this paper, the gasket used for research was judged to be a material with strong nonlinearity, so a conservative value of 0.01 was assigned to $w$ to attribute more significance to complexity than sparsity. The model with the minimum cost, an 8-parameter model (Case 2), was chosen. Equation (7) represents the model, where ${k}_{0}$ and ${c}_{0}$ signify linear stiffness and linear damping coefficients, respectively. Additionally, ${k}_{1}$ ~ ${k}_{n}$ and ${c}_{1}$~ ${c}_{n}$ denote nonlinear stiffness and nonlinear damping coefficients, respectively. Table 4 provides the specific values for these coefficients.

**Table 4:** Regression modeling for Case 2.

Coefficient	Value (unit: g, mm, s)
${k}_{0}$	7.50 $\times {10}^{6}$
${k}_{1}$	− 6.48 $\times {10}^{4}$
${k}_{2}$	7.26 $\times {10}^{2}$
${k}_{3}$	6.32 $\times 10$
${c}_{1}$	1.96 $\times {10}^{3}$
${c}_{2}$	− 1.25 $\times {10}^{3}$
${c}_{3}$	− 3.09 $\times 10$
${c}_{4}$	1.90 $\times 10$

7 $$F-m\ddot{x}=\left({k}_{0}+{k}_{1}\omega {x}^{3}+{k}_{2}{\omega }^{2} x+{k}_{3}{\omega }^{2}{x}^{3} \right)x+(c\_1 \omega +{c}_{2}\omega {x}^{2}+{c}_{3}{\omega }^{2}+{c}_{4}{\omega }^{2}{x}^{2})\dot{x}$$

Unlike Case 2, where we lacked linearity insights, Case 3 incorporates these insights by assigning weights to the linear terms $x$ and $\dot{x}$. Other than this, the nonlinearity was regressed using the same method as in Case 2. This means ${L}_{1}$ cost function and sparsity cost, and as a result of constructing regression model using this approach, the number of parameters was obtained to be the same. Consequently, we obtained another sparse model, specifically identified as a sparse regression model with linear term weights (Case 3), expressed in Eq. (8). In alignment with the Case 2 model, ${k}_{0}$ and ${c}_{0}$ denote linear stiffness and linear damping coefficients, while ${k}_{1}$~ ${k}_{n}$ and ${c}_{1}$~${c}_{n}$ signify nonlinear stiffness and nonlinear damping coefficients. Precise values of these coefficients are referred to Table 5.

**Table 5:** Regression modeling for Case 3.

Coefficient	Value (unit: g, mm, s)
${k}_{0}$	8.40 $\times {10}^{6}$
${k}_{1}$	− 3.02 $\times {10}^{5}$
${k}_{2}$	7.01 $\times {10}^{3}$
${c}_{0}$	7.92 $\times {10}^{4}$
${c}_{1}$	− 1.87 $\times {10}^{3}$
${c}_{2}$	− 3.60 $\times {10}^{3}$
${c}_{3}$	1.13 $\times 10$
${c}_{4}$	8.44 $\times 10$

8 $$F-m\ddot{x}=\left({k}_{0}+{k}_{1}\omega {x}^{3}+{k}_{2} {\omega }^{2} {x}^{3} \right)x+({c}_{0}+{c}_{1} \omega +{c}_{2} \omega {x}^{2}+{c}_{3} {\omega }^{3}+{c}_{4} {\omega }^{2} {x}^{2})\dot{x}$$

### Validation experiment result

**Figure 14:**
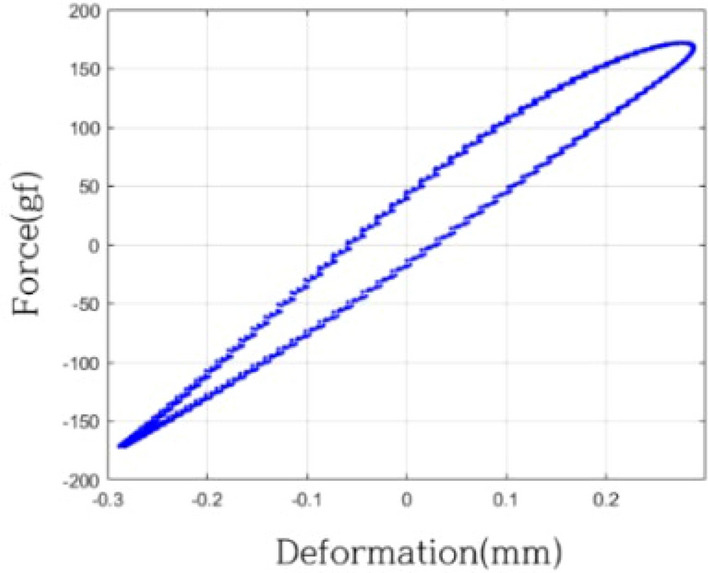
Deformation-force curve of validation system (8.2 Hz).

Validation experiments covered six cases with vibration frequencies ranging from 3 to 12 Hz (3.2 Hz, 4.2 Hz, 6.2 Hz, 8.2 Hz, 8.3 Hz, 11.8 Hz). Frequencies below 10 Hz fall within the regression range, while those above 10 Hz are non-regression frequencies. Displacement, measured within the − 0.3 mm to 0.3 mm range, corresponds to strain ($\varepsilon$) magnitudes from − 0.03 to 0.03. In Fig. 14, the displacement-force curve for the validation system under 8.2 Hz excitation confirms nonlinearity, attributed to variations in damping forces during expansion and contraction.

**Figure 15:**
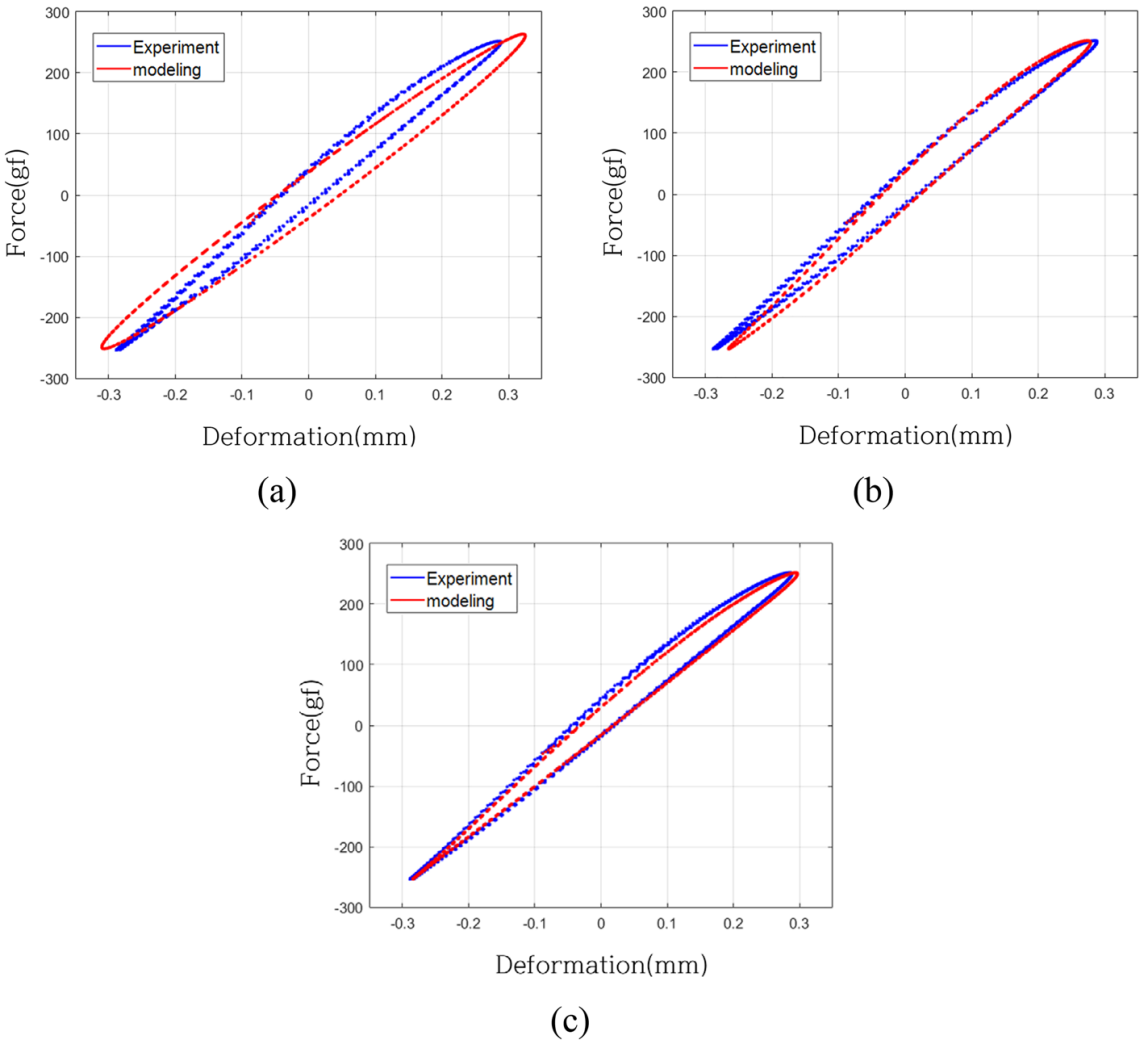
Deformation-force curve of validation system and modeling (8.2 Hz): (**a**) Case 1, (**b**) Case 2, (**c**) Case 3.

Figure 15 provides a comparative analysis of displacement-force curves for both the validation system and the regression model, shedding light on the similarity between the predicted and actual system behaviors. In Fig. 15a, the validation system is juxtaposed with the linear model (Case 1), revealing noticeable disparities in curve slope and the location of the maximum force. Figure 15b showcases the model without the weighting of linear terms (Case 2), adeptly capturing the curve's slope but diverging at the point of maximum force. Figure 15c illustrates the model with the weighting of linear terms (Case 3), presenting the closest match to the maximum force point and accurately mirroring the curve's slope.

**Figure 16:**
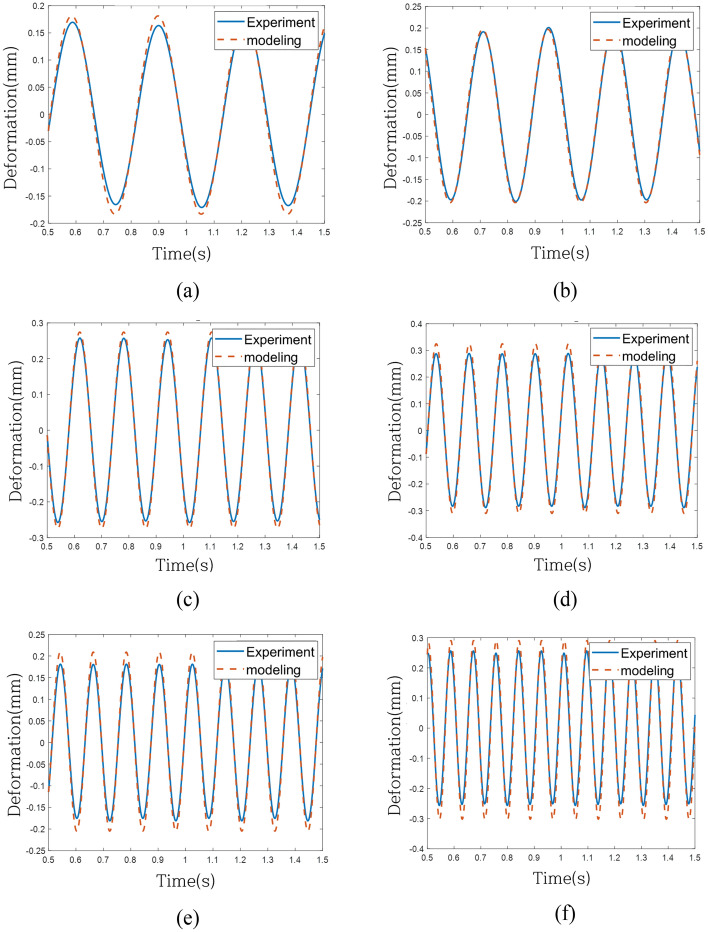
Time-domain response of experiment and modeling (Case 1): (**a**) 3.2 Hz, (**b**) 4.2 Hz, (**c**) 6.2 Hz, (**d**) 8.2 Hz, (**e**) 8.3 Hz and (**f**) 11.8 Hz.

**Figure 17:**
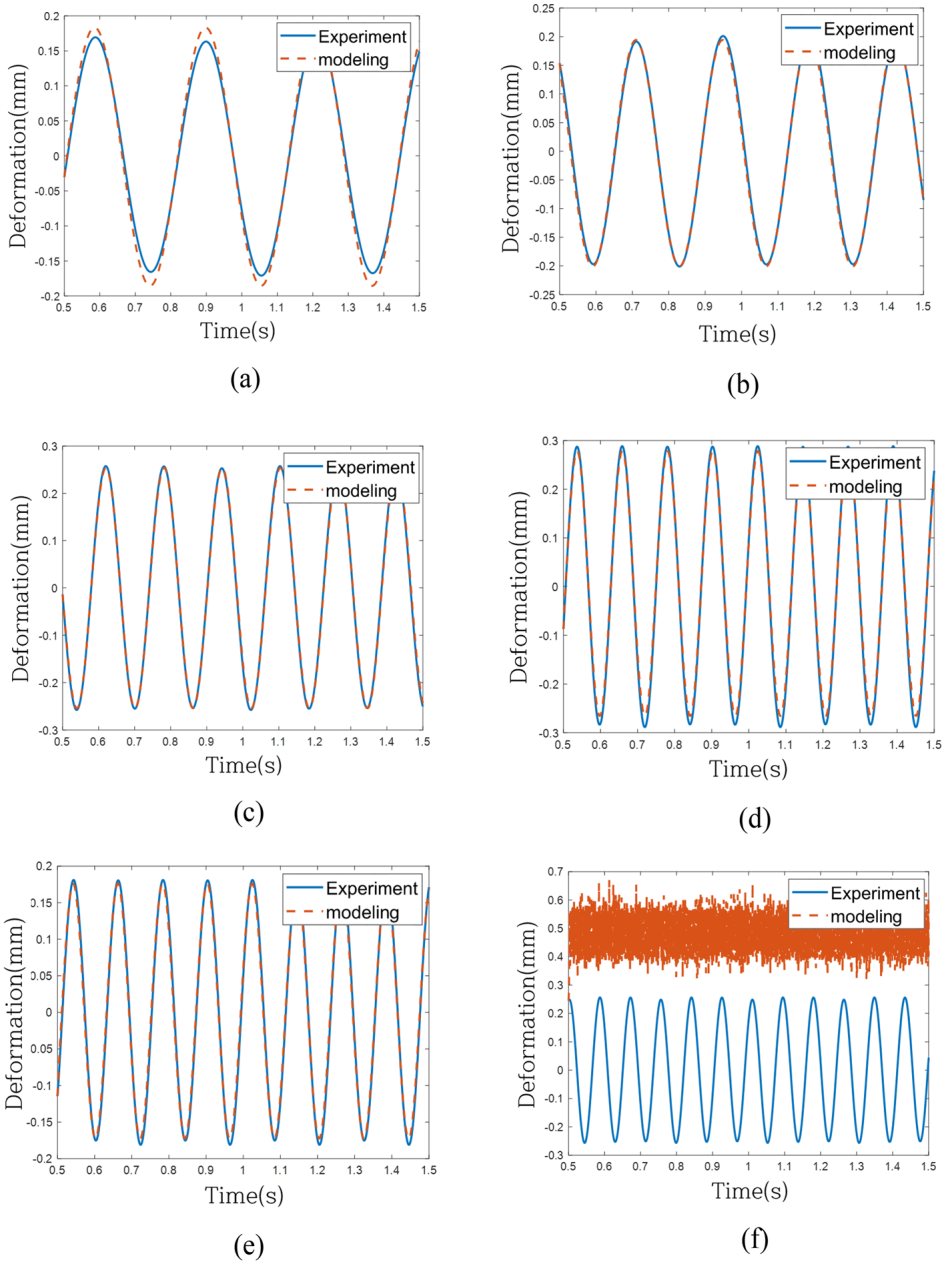
Time-domain response of experiment and modeling (Case 2): (**a**) 3.2 Hz, (**b**) 4.2 Hz, (**c**) 6.2 Hz, (**d**) 8.2 Hz, (**e**) 8.3 Hz and (**f**) 11.8 Hz.

**Figure 18:**
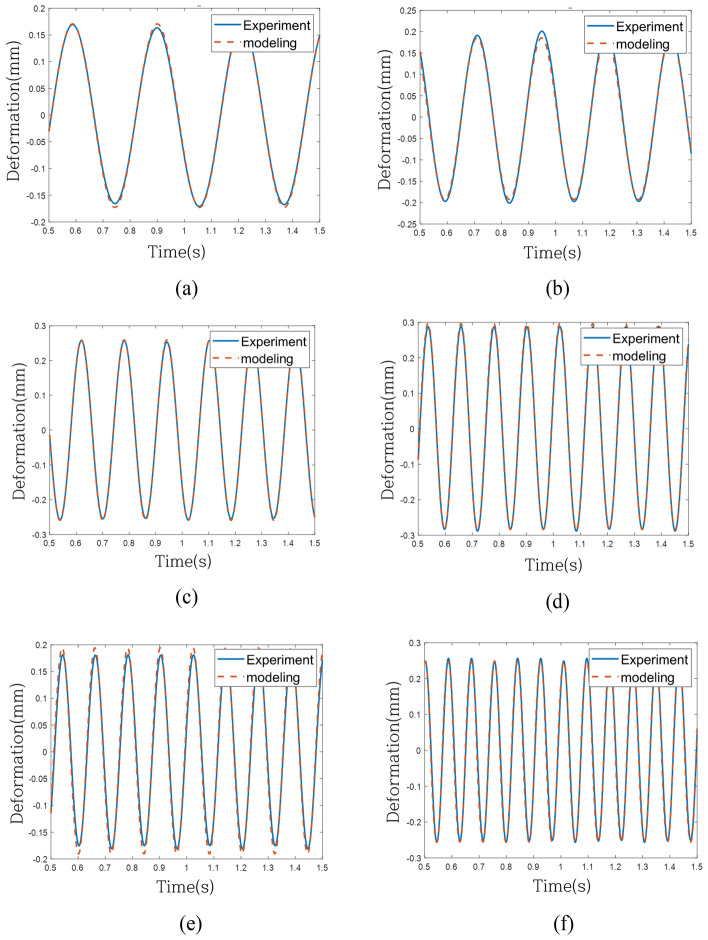
Time-domain response of experiment and modeling (Case 3): (**a**) 3.2 Hz, (**b**) 4.2 Hz, (**c**) 6.2 Hz, (**d**) 8.2 Hz, (**e**) 8.3 Hz and (**f**) 11.8 Hz.

To assess the predictive model's performance under the same excitation force as the validation system, individual comparisons were conducted in the time domain across 6 frequencies, as depicted in Fig. 16 for the linear model (Case 1). Although the linear model exhibited convergence across all frequencies, its effectiveness in tracking the actual behavior was limited. Figure 17 depicts the response of the model without the weighting of linear terms (Case 2), effectively tracking behavior within the regression frequency range (1 Hz to 10 Hz) but experiencing divergence beyond 10 Hz. Finally, Fig. 18 portrays the response of the model with weighting linear terms (Case 3), demonstrating convergence across all validation frequencies and proving most effective in accurately tracking the actual behavior among the three models.

The peak-to-peak ($\Delta x$) is calculated as the difference between the maximum and minimum values of signal $x$, as shown in Eq. (9).

9 $$\Delta x={x}_{max}-{x}_{min}$$

Additionally, the peak-to-peak error (*ε*_*p*_) is calculated by dividing the difference between the predicted model and the experiment peak-to-peak by the experiment peak-to-peak, using the same method as Eq. (10).

10 $${\varepsilon }_{p}=\left|\frac{{\varepsilon }_{p,test}-{\varepsilon }_{p,prediction}}{{\varepsilon }_{p,test}}\right|$$

**Figure 19:**
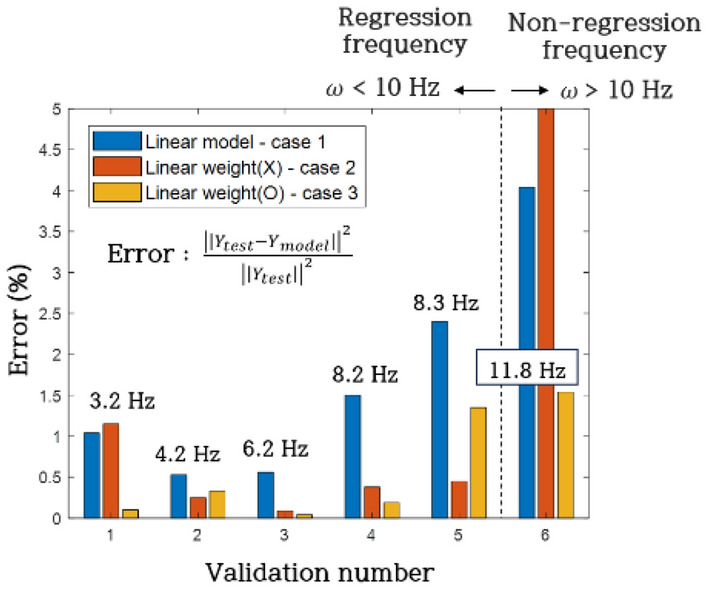
Least square error of regression model and experiment.

The responses of the linear model (Case 1) and sparse regression models (Case 2, 3) were individually compared with the validation system, and errors were computed as part of the evaluation process. Error calculations were performed using the least squares method, which involved comparing response magnitudes (peak to peak). The validation results, obtained from six distinct verification scenarios corresponding to excitation frequencies of 3.2 Hz, 4.2 Hz, 6.2 Hz, 8.2 Hz, 8.3 Hz, and 11.8 Hz, are presented in Fig. 19 and Table 6 based on the least squares method. Each validation number (1–5) aligned with regression frequency domains, while validation number 6 represented a non-regression frequency. The Case 1 model demonstrated convergence for all validation numbers (1–6), yielding an average least squares error of 1.68%. The Case 2 model exhibited convergence for validation numbers 1 to 5 but diverged at 6, with an average least squares error of 0.46% for validation numbers 1 to 5. Conversely, the Case 3 model demonstrated convergence for all validation numbers (1–6), registering an average least squares error of 0.59%.

**Table 6:** Least square error of regression model and experiment.

Validation number	Case 1 (%)	Case 2 (%)	Case 3 (%)
1	1.04	1.15	0.1
2	0.53	0.25	0.33
3	0.56	0.00088	0.00041
4	1.5	0.38	0.19
5	2.4	0.45	1.35
6	4.04	Divergence	1.54
Mean	1.68	0.46	0.59

**Figure 20:**
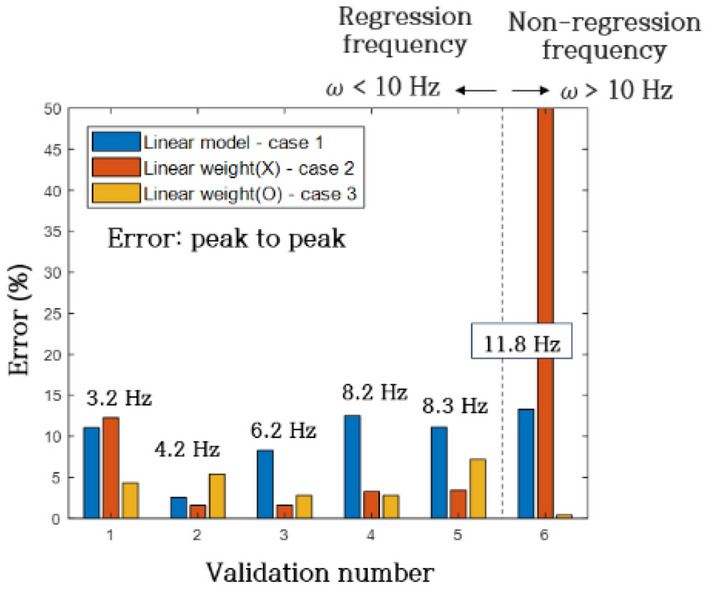
Peak to peak error of regression model and experiment.

In Fig. 20 and Table 7, a comprehensive comparison of response magnitudes (peak to peak) between the regression models and actual responses is presented. The validation numbers and their corresponding frequencies align with the least squares error graph (Fig. 19). The Case 1 model displayed an average error of 9.8% across validation numbers 1 to 6. Meanwhile, the Case 2 model diverged at frequency 6, resulting in an average error of 4.41% for validation numbers 1 to 5. The Case 3 model exhibited an average error of 3.80% across all validation numbers 1 to 6. These findings provide a detailed assessment of the predictive accuracy and robustness of the regression models under varied verification scenarios.

**Table 7:** Peak to peak error of regression model and experiment.

Validation number	Case 1 (%)	Case 2 (%)	Case 3 (%)
1	11.04	12.27	4.29
2	2.55	1.57	5.41
3	8.30	1.58	2.78
4	12.5	3.24	2.78
5	11.11	3.43	7.18
6	13.28	Divergence	0.4
Mean	9.80	4.41	3.80

## Discussion and conclusion

This research emphasizes the use of regression methods to identify the dynamic properties of sponge-like materials characterized by substantial nonlinearity. Proficient regression models have been introduced to accurately predict the response of nonlinear systems. This involves integrating dynamical background knowledge with the conventional regression approach, SINDy, and imposing physical constraints to construct the regression model. This is achieved by fixing weights on linear parameters. In this paper, Cases 1–2 serve as control groups, while the regression model obtained through the proposed method is named Case 3. Additionally, the construction of the regression model in this paper involves tasks such as parameter regularization and extracting sparse models through an algorithm employing L1 cost functions, accompanied by error histograms to provide visual aid. Meanwhile, the sources of errors between actual behavior and regression models are outlined below.

1. Imperfections in Measurement Equipment: In this study, errors in the sampling rate, resolution of sensors, and the small mass of the vibration ground contributed to imperfections in measurement equipment. These issues can interfere with fixed boundary conditions, particularly in high-frequency vibrations, potentially introducing noise.
2. Limited Frequency Range: Due to imperfections in the measurement equipment, the data used for constructing the regression model is limited to frequencies below 10 Hz. This limitation results in insufficient information about the physical behavior at frequencies above 10 Hz, potentially leading to errors or divergence in the regression model in the higher frequency range.
3. Modal aliasing: Nonlinear systems exhibit more complex behavior than linear systems, characterized by amplitude dependence, frequency dependence, and the superposition of harmonic and subharmonic modes. In this paper, we attempted to address these complexities using high sampling frequencies. However, employing this strategy alone has limitations when dealing with strong nonlinear effects. To improve these problems, nonlinear system regression and modal parameter analysis can be helpful.

In this research paper, we meticulously examined the regression-based predictive models derived through identification experiments of the actual system behavior to validate the proposed methodology. The linear model(Case 1) serving as a control, successfully avoided divergence issues but fell short in accurately replicating the nonlinear characteristics of the system. In contrast, both the Case 2 and Case 3 effectively tracked responses within the regression frequency domain. However, the Case 2 model faced divergence issues outside the regression frequency domain, while model Case 3 consistently tracked responses. This indicates that the nonlinear model with physical constraints (Case 3) possesses broader applicability as a predictive model compared to unconstrainted nonlinear model (Case 2).

Each regression model can be summarized as follows:

1. Case 1: A linear model derived through linear regression. While this model converges for all frequencies, it fails to effectively predict the system's response.
2. Case 2: A sparse regression model without weighting the linear candidate function terms. This model effectively predicts the system's response within the regression frequency range. However, in the non-regression frequency range, the predicted response diverges.
3. Case 3: A sparse regression model with weights on the linear candidate function terms. This model successfully tracks the response in all frequency ranges and does not exhibit divergence issues. Therefore, this model is considered the most general and effective.

Data regression techniques typically exhibit high accuracy within the regression domain but lower accuracy outside of it, leading to frequent data scarcity issues. Additionally, this paper encountered difficulty in appropriately identifying systems outside the regression domain when using conventional methods alone, as evident in Fig. 17f of our study. To address this issue, we partially employed dynamic knowledge and weighted linear parameters, as in Case 3 of our research. As a result, we achieved higher accuracy not only in estimating models outside the regression domain but also in modeling within it. Constructing sparse models with physical constraints effectively captures the dynamic characteristics of nonlinear systems, providing insights into the complexity of real systems. This insight is facilitated by assigning weights to linear terms, offering understanding into the intricate phenomena of real systems. This methodology is applicable to dynamic analysis or prediction of joint components with nonlinear materials, like polymers, and holds potential for diverse applications in mechanical systems. The proposed modeling technique offers a valuable tool for addressing ambiguous boundary conditions and nonlinearity in the real world, contributing to the efficient design and performance enhancement of mechanical joint components.

## Data Availability

The datasets used and/or analyzed during the current study are available from the corresponding author on reasonable request.
